# Supplementary material for: Estimating the contribution of CD4 T cell subset proliferation and differentiation to HIV persistence
Source: Nat Commun. 2023 Oct 2;14:6145. doi: 10.1038/s41467-023-41521-1 (PMC10545742; doi:10.1038/s41467-023-41521-1)
Supplement: Supplementary file 1 — Supplementary Information [file 41467_2023_41521_MOESM1_ESM.pdf]

Supplementary Information

Estimating the contribution of CD4 T cell proliferation and differentiation to HIV persistence

Daniel B Reeves*, Charline Bacchus-Souffan*, Mark Fitch, Mohamed Abdel-Mohsen, Rebecca Hoh, Haelee Ahn, Mars Stone, Rick Hecht, Jeff Martin, Steven G Deeks, Marc K Hellerstein, Joseph M McCune, Joshua T Schiffer**, Peter W Hunt**

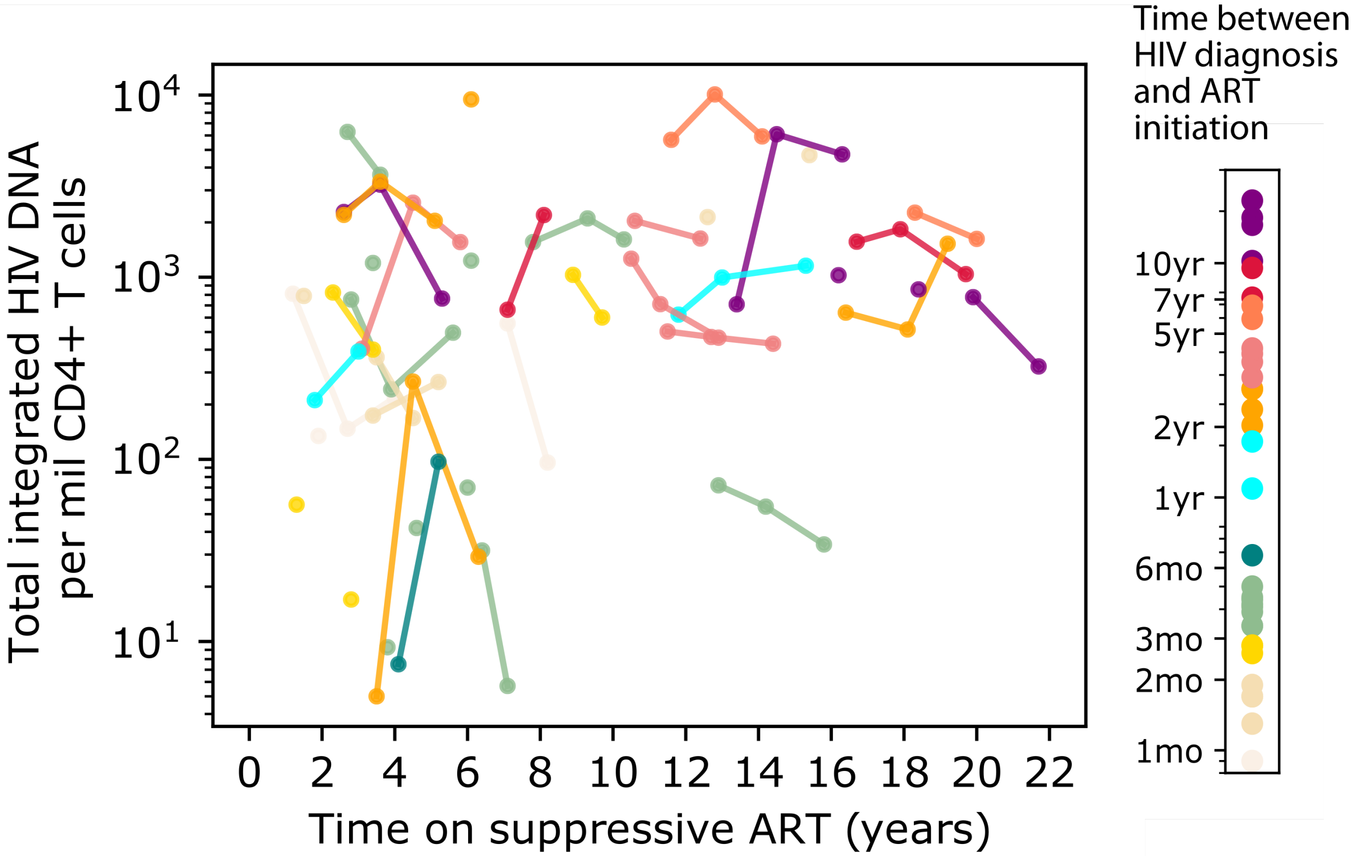

Supplementary Figure 1. Total longitudinal HIV DNA in 37 PWH, arranged according to time on ART and distinguished by approximate time of HIV infection before initiation of ART.
HIV DNA per million CD4 T cells (sum of subsets) for each participant illustrating duration of ART and time between HIV diagnosis and ART initiation (color). Neither variable was significantly predictive of total HIV DNA levels.

Supplementary Table 1. **Study participant characteristics**.
All participants are male and had viral loads <40 copies/mL upon study initiation.

ID	Age decade (years)	Estimated time between HIV acquisition and ART initiation (months)	ART duration at study initiation (years)	CD4 count at study initiation (cells/μL blood)	CD4 nadir (cells/μL blood)	CD4/CD8 ratio at study initiation
1	40	0.9	1.2	841	468	0.9
2	60	4.2	2.8	341	252	0.7
3	30	1.7	1.5	899	518	1.5
4	30	4.5	3.4	532	486	1.3
5	50	1.9	3.4	1163	618	1.9
6	40	4.1	2.7	637	509	1.0
7	20	1.7	3.5	787	341	1.7
8	40	2.8	1.3	445	311	1.0
9	30	5	3.8	433	251	1.2
10	20	2.6	2.3	1290	791	1.4
11	30	0.1	1.9	673	437	1.0
12	30	0.7	7.1	513	352	1.2
13	50	3.4	12.9	739	442	1.3
14	40	4.4	6.4	341	203	1.0
15	50	1.3	12.6	469	165	0.8
16	30	3.4	7.8	697	391	1.1
17	40	2.8	8.9	880	478	1.1
18	30	3.9	4.6	518	202	1.2
19	50	34.9	4.5	573	311	1.2
20	30	45.5	3.1	637	357	1.1
21	30	24.4	3.5	626	480	1.3
22	30	20.8	1.8	902	664	1.1
23	20	6.8	4.1	582	229	2.3
24	50	187.7	2.6	432	335	1.7
25	50	34.8	2.6	468	393	1.0
26	60	85.5	16.7	383	132	1.1
27	50	222	13.4	377	300	0.6
28	60	122.5	18.8	687	13	1.1
29	50	39.1	11.5	523	2	0.7
30	50	69.4	17.0	747	10	0.4
31	50	13.1	11.8	486	280	1.3
32	60	114.5	7.1	437	54	1.4
33	60	49.3	10.6	570	207	0.8
34	60	28.5	16.4	486	96	1.2
35	50	79	11.6	613	99	1.0
36	60	175	16.2	667	400	1.2
37	40	51.9	10.5	474	129	0.6

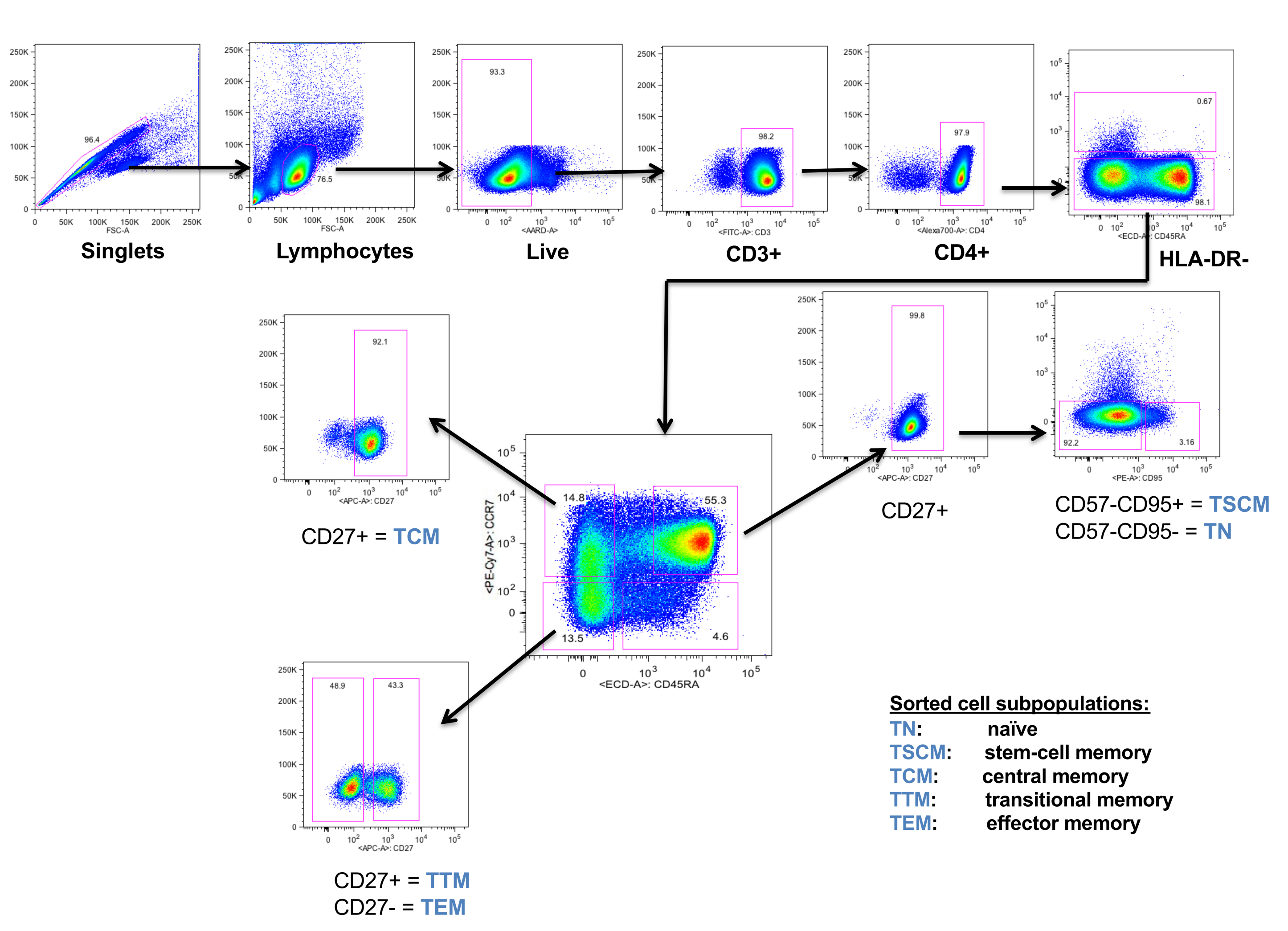

Supplementary Figure 2. Cell sorting strategy for CD4 T cell subsets. Representative flow plots from a single participant in the study showing the sorting strategy used to isolate the various CD4 T cell subsets. Once resting (HLA-DR-) CD4+ T cells were selected, we used the extracellular markers CD45RA, CCR7, CD27, CD57 and CD95 to distinguish the following cell subsets: T_N, T_{SCM}, T_{CM}, T_{TM} and T_{EM}. The terminally-differentiated (T_{TD}) population (CD45RA+ CCR7-, bottom right pink box in central panel) was not reported here as variable contamination of T_N was found in this population as described previously[23].

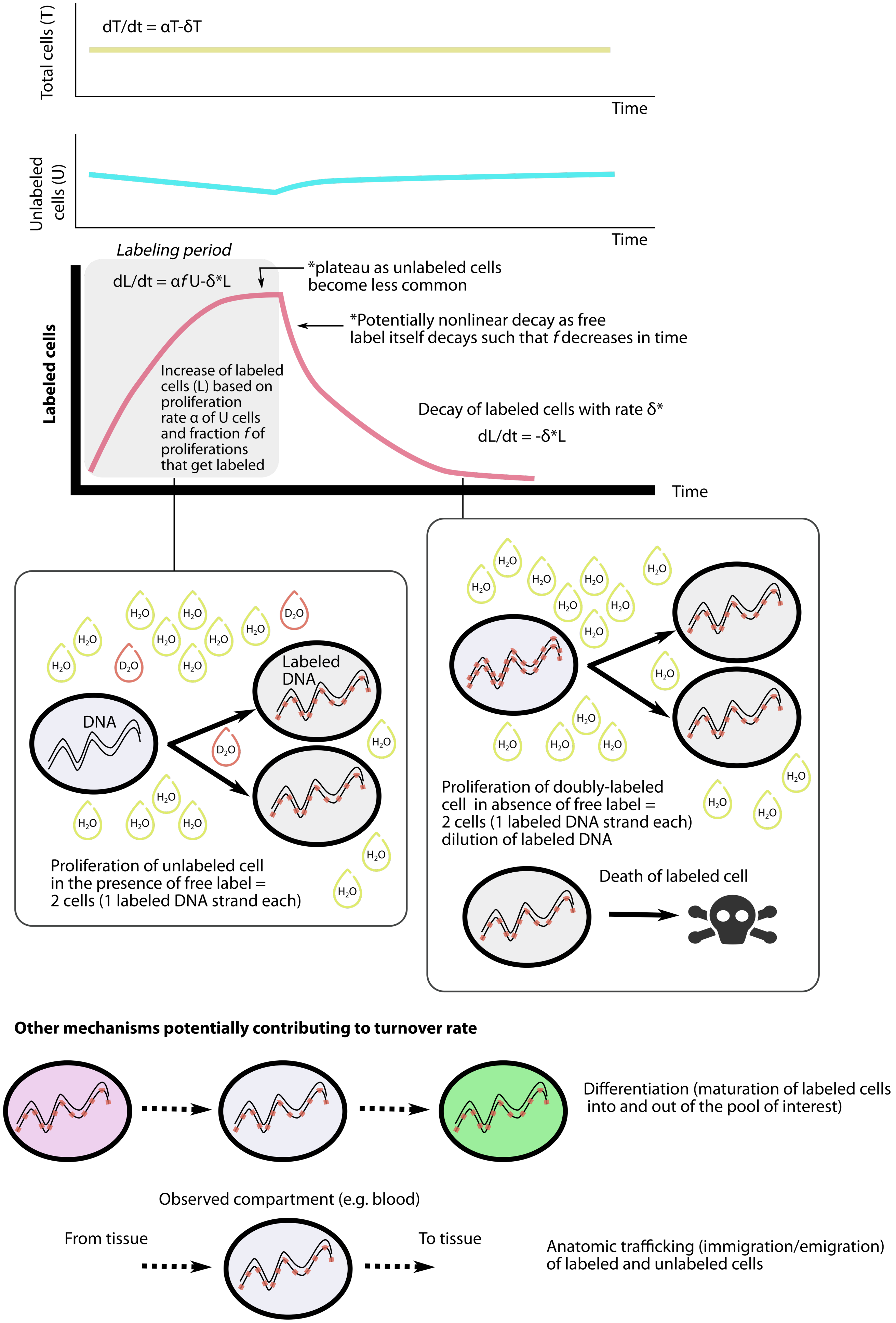
Total cells (T)
dT/dt = αT-δT
Time
Unlabeled cells (U)
Time
Labeling period
dL/dt = αf U-δ*L
*plateau as unlabeled cells become less common
*Potentially nonlinear decay as free label itself decays such that f decreases in time
Labeled cells
Increase of labeled cells (L) based on proliferation rate α of U cells and fraction f of proliferations that get labeled
Decay of labeled cells with rate δ*
dL/dt = -δ*L
Time
H2O
D2O
Labeled DNA
DNA
Proliferation of unlabeled cell in the presence of free label = 2 cells (1 labeled DNA strand each)
Proliferation of doubly-labeled cell in absence of free label = 2 cells (1 labeled DNA strand each) dilution of labeled DNA
Death of labeled cell
Other mechanisms potentially contributing to turnover rate
Differentiation (maturation of labeled cells into and out of the pool of interest)
Observed compartment (e.g. blood)
From tissue
To tissue
Anatomic trafficking (immigration/emigration) of labeled and unlabeled cells

Supplementary Figure 3. Schematic and cartoon of labeling study. The top three cartoon graphics illustrate time-varying levels of total cells (yellow), unlabeled cells (blue), and labeled cells (red). The fraction of labeled cells L/T is used to infer turnover rate. Several assumptions are made. First, total cells in each subset are assumed to stay constant over the 45-day labeling period. Unlabeled cells may decrease slightly (3% in our study), so this is approximately negligible in the dynamic system. The remaining graphics illustrate cell processes occurring in a time slice during labeling (left) and after labeling ceases (right). During the labeling period, deuterium -- D_2O (red drops) vs normal H_2O (yellow drops) -- is available and can be incorporated into the genome of dividing cells to make labeled DNA. A single unlabeled cell dividing results in 2 cells carrying a single labeled DNA strand each. After administration ceases, proliferation of cells then results in new cells without deuterium, and this coupled with the clearance of labeled cells results in a dilution of the labeled fraction. A doubly labeled cell dividing results in 2 cells carrying a single labeled DNA strand each. There are other possible mechanisms (dashed arrows) that could potentially contribute to turnover kinetics, including differentiation of already labeled cells into a certain subset, or differentiation out of the subset of interest. In addition, immigration/migration are assumed to be balanced and ignorable, but this theoretically could influence dynamics.

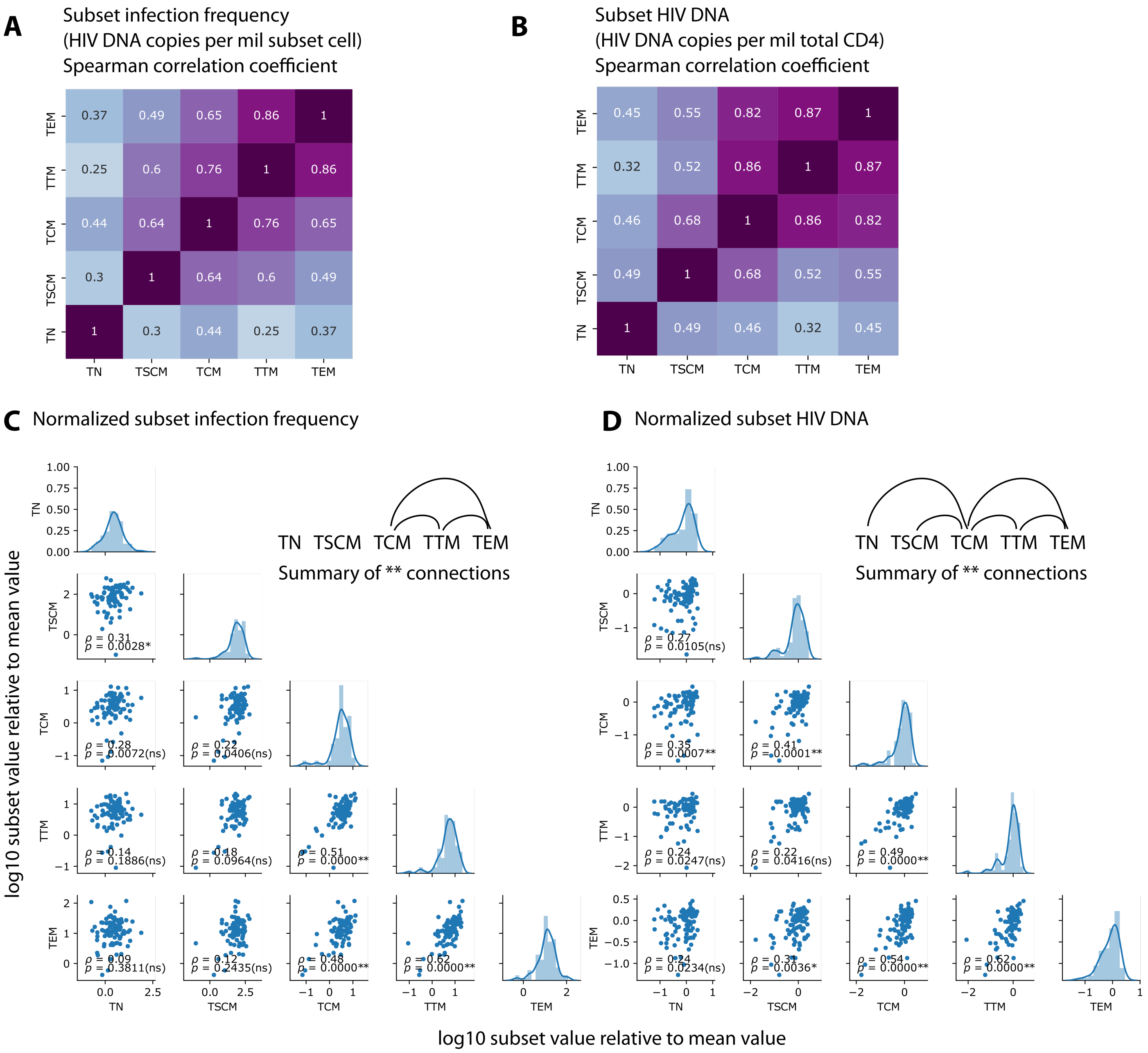

Supplementary Figure 4. Correlations among subset HIV DNA levels across and within individuals. A) Spearman correlations between the time-averaged values of subset frequency over time $\langle \mathbf{f_X} \rangle_t$. B) Spearman correlations between the time-averaged values of the subset HIV DNA $\langle \mathbf{H_X} \rangle_t$. C) Spearman correlations between normalized subset frequency data (normalized to time-averaged values $\mathbf{\hat{f}_X(t)} = \mathbf{f_X(t)}/\langle \mathbf{f_X} \rangle_t$ across all individuals and time points (for instance, correlation between TCM and TEM follows $\mathbf{\hat{f}_{CM}(t)} \sim \mathbf{\hat{f}_{EM}(t)}$). D) Spearman correlations between normalized subset HIV DNA levels across all individuals and time points (also normalized to time-averaged values). For C and D, data are visualized on log10 scale, and plot insets provide correlation coefficients (ρ) and p-values. P-values were characterized as not significant (ns) if $p>0.005$, significant (*) if $p<0.005$, and strongly significant (**) if $p<0.001$ based on a Bonferroni correction due to 10 comparisons in each of A and B. Strongly significant correlations are summarized for each data type in the line diagram to the top right of the scatter plots. TCM, TTM, and TEM were significantly related for both data types.

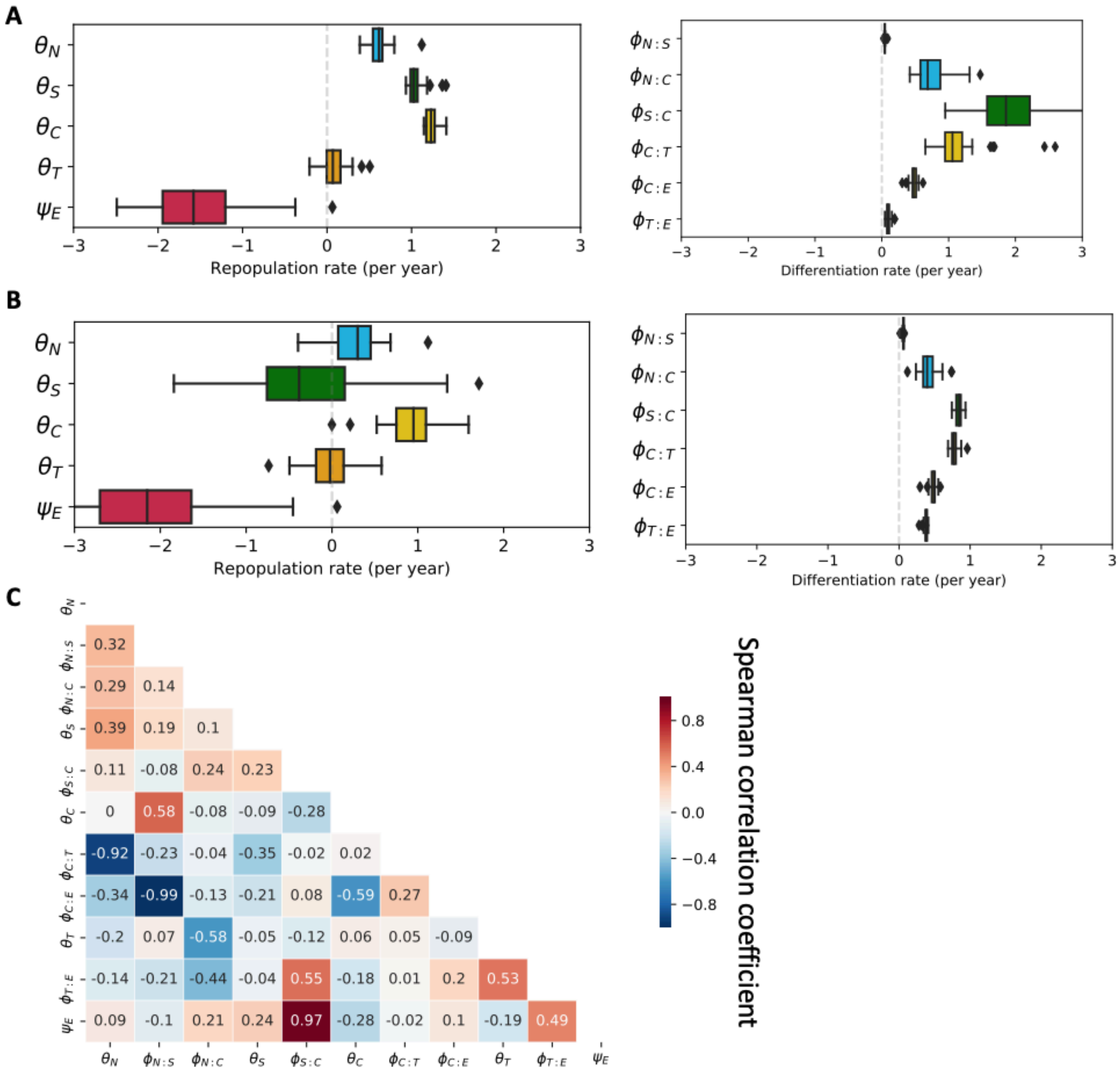

Supplementary Figure 5. Estimated parameter values from the best model. Based on the observation of very high differentiation rates (>100% HIV DNA per year), we constrained the parameter space available for the fitting algorithm. A) The initial values from the top ranked model. B) The constrained values from the second best model, but with more biologically plausible values. Box plots indicate median (center), interquartile range (box) and 1.5x interquartile range (fliers), outliers are denoted by diamond. C) Correlations (Spearman coefficient) among estimates of the parameters across all individuals.

Supplementary Table 2. Population parameters, variation across individuals, and error of estimate for best model. Note model noise is included with the equation $y_i = T_i + b_i T_i \epsilon$ where ϵ is a normal distribution with zero mean and unit variance and T_i is the level of subset i.

Category [unit]	Symbol	Population parameter value	Residual squared error of estimate (%)	Std deviation of participant values
Initial condition	$H_N(0)$	29.03	28	1.63
[HIV DNA per	$H_S(0)$	2.37	27	1.62
mil CD4+ T cells]	$H_C(0)$	163.70	29	1.70
	$H_T(0)$	89.80	28	1.67
	$H_E(0)$	65.81	30	1.80
Net proliferation-death	θ_N	0.30	95	0.44
"net" rate [1/year]	θ_S	-0.40	270	1.03
	θ_C	0.94	33	0.45
	θ_T	-0.07	171	0.40
TEM net + differentiation out rate [1/year]	Ψ_E	-2.23	28	1.23
Differentiation rate	$\varphi_{N:S}$	0.06	22	0.02
[1/year]	$\varphi_{N:C}$	0.40	67	0.26
	$\varphi_{S:C}$	0.85	123	0.25
	$\varphi_{C:T}$	0.77	25	0.16
	$\varphi_{C:E}$	0.48	41	0.14
	$\varphi_{T:E}$	0.38	71	0.13
Estimated variation of	b_N	0.62	14	
data (noise) [unitless]	b_S	0.35	21	
	b_C	0.51	13	
	b_T	0.42	17	
	b_E	0.43	19	

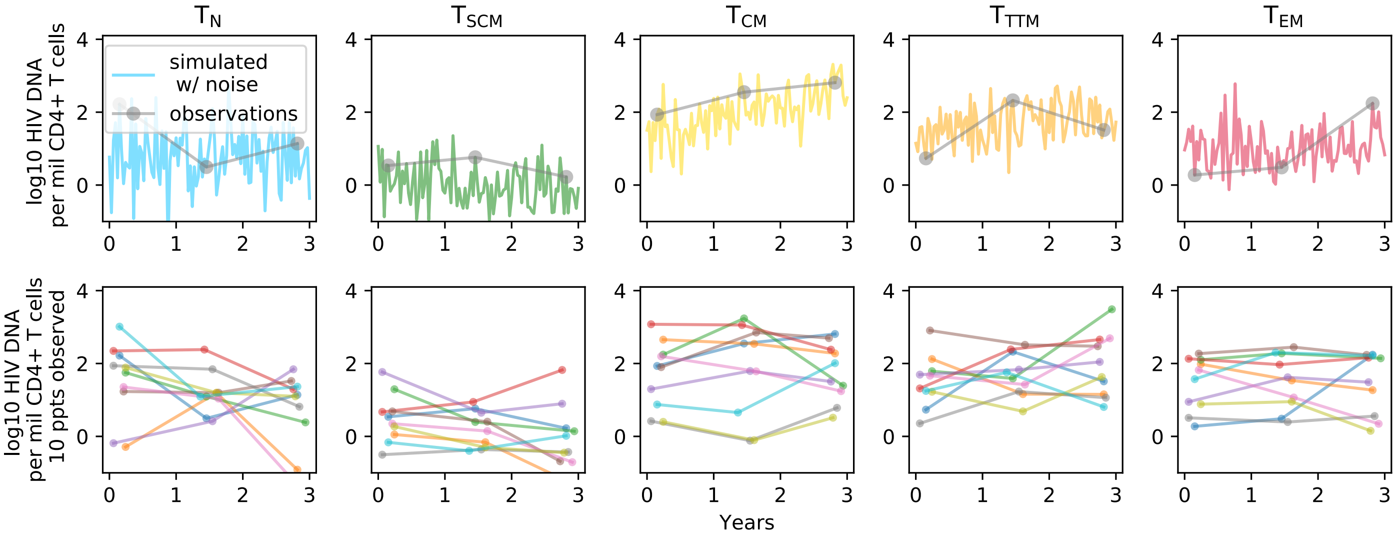

Supplementary Figure 6. Simulating linear differentiation model to assess model selection after sampling. Top: A single participant example of simulated data from the model with linear differentiation (and noise) for each subset (colored jagged lines) and random samples around 0, 1.5, and 3 years (with 10 weeks of variability). Bottom: 10 example participant observed trajectories (colors) for each subset above to illustrate heterogeneity of sparsely sampled data.

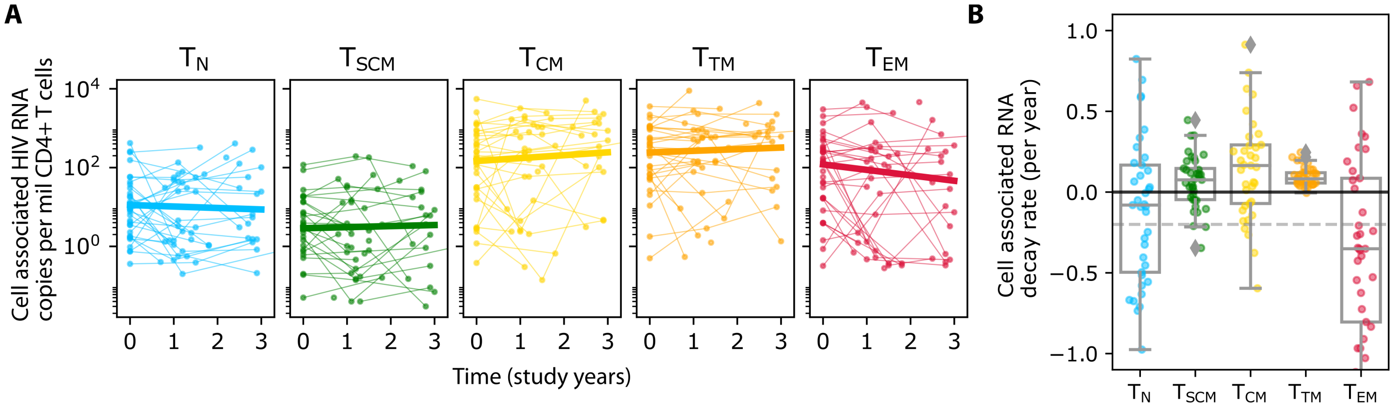

Supplementary Figure 7. Cell-associated HIV RNA kinetics. A) Levels in each participant, dots are observations and lines connect dots to help visualize individual trajectories. Solid line is best fit population model. B) Decay rate for each subset, dots indicate values for each participant. Box plots indicate median (center), interquartile range (box) and 1.5x interquartile range (fliers), outliers are denoted by diamond.
