# Supplementary material for: Estimating the contribution of CD4 T cell subset proliferation and differentiation to HIV persistence
Source: Nat Commun. 2023 Oct 2;14:6145. doi: 10.1038/s41467-023-41521-1 (PMC10545742; doi:10.1038/s41467-023-41521-1)
Supplement: Supplementary file 3 — Description of additional Supplementary files [file 41467_2023_41521_MOESM3_ESM.pdf]

Description of Additional Supplementary Files Document

Supplementary Movie 1. Model simulation. Simulation of best model with population parameters for a typical 1 day, 1 week, 1 month, and 1 year of ART. Populations of integrated HIV DNA in each subset constantly proliferate, die, and differentiate into other subsets. However, net levels in each subset remain mostly constant, with a slight decay in more mature subsets that can only be seen over years of ART.

Supplementary Data 1: Individual initial conditions and rates/parameter values for each participant taken from the pNLME mode output by Monolix. Can be used to resimulate any individual trajectory.
