# Supplementary material for: Estimating the contribution of CD4 T cell subset proliferation and differentiation to HIV persistence
Source: Nat Commun. 2023 Oct 2;14:6145. doi: 10.1038/s41467-023-41521-1 (PMC10545742; doi:10.1038/s41467-023-41521-1)
Supplement: Supplementary file 6 — Reporting Summary [file 41467_2023_41521_MOESM6_ESM.pdf]

nature portfolio

Corresponding author(s): Daniel Reeves

Last updated by author(s): Aug 15, 2023

Reporting Summary

Nature Portfolio wishes to improve the reproducibility of the work that we publish. This form provides structure for consistency and transparency in reporting. For further information on Nature Portfolio policies, see our Editorial Policies and the Editorial Policy Checklist.

Statistics

For all statistical analyses, confirm that the following items are present in the figure legend, table legend, main text, or Methods section.

n/a	Confirmed	
☐	☒	The exact sample size (*n*) for each experimental group/condition, given as a discrete number and unit of measurement
☐	☒	A statement on whether measurements were taken from distinct samples or whether the same sample was measured repeatedly
☐	☒	The statistical test(s) used AND whether they are one- or two-sided<br>*Only common tests should be described solely by name; describe more complex techniques in the Methods section.*
☐	☒	A description of all covariates tested
☐	☒	A description of any assumptions or corrections, such as tests of normality and adjustment for multiple comparisons
☐	☒	A full description of the statistical parameters including central tendency (e.g. means) or other basic estimates (e.g. regression coefficient) AND variation (e.g. standard deviation) or associated estimates of uncertainty (e.g. confidence intervals)
☐	☒	For null hypothesis testing, the test statistic (e.g. *F*, *t*, *r*) with confidence intervals, effect sizes, degrees of freedom and *P* value noted<br>*Give P values as exact values whenever suitable.*
☒	☐	For Bayesian analysis, information on the choice of priors and Markov chain Monte Carlo settings
☒	☐	For hierarchical and complex designs, identification of the appropriate level for tests and full reporting of outcomes
☐	☒	Estimates of effect sizes (e.g. Cohen's *d*, Pearson's *r*), indicating how they were calculated

Our web collection on statistics for biologists contains articles on many of the points above.

Software and code

Policy information about availability of computer code

Data collection	Code is freely available here: https://github.com/dbrvs/HOPE-modeling
Data analysis	Analysis code is freely available here: https://github.com/dbrvs/HOPE-modeling

For manuscripts utilizing custom algorithms or software that are central to the research but not yet described in published literature, software must be made available to editors and reviewers. We strongly encourage code deposition in a community repository (e.g. GitHub). See the Nature Portfolio guidelines for submitting code & software for further information.

Data

Policy information about availability of data

All manuscripts must include a data availability statement. This statement should provide the following information, where applicable:

- Accession codes, unique identifiers, or web links for publicly available datasets
- A description of any restrictions on data availability
- For clinical datasets or third party data, please ensure that the statement adheres to our policy

All data used in the study are freely available here: https://github.com/dbrvs/HOPE-modeling

Human research participants

Policy information about studies involving human research participants and Sex and Gender in Research.

Reporting on sex and gender	Biological sex for all participants is provided in Supplementary Table 1
Population characteristics	Study participant characteristics are provided in Supplementary Table 1
Recruitment	Participants were enrolled into the study if they were 18-69 years old; and if they were virally suppressed while taking antiretroviral therapy (ART) for at least one year; and if females of childbearing potential had a negative serum pregnancy test at screening and agreed to use a double-barrier method of contraception throughout the study period. Enrolled participants were then categorized into four groups: ART initiated less than 100 days after acute infection and maintained for 1-3 years; ART initiated less than 100 days after acute infection and maintained for at least 7 years; ART initiated at least 3 years after acute infection and maintained for 1-3 years; and ART initiated at least 3 years after acute infection and maintained for at least 7 years. Individuals with ART initiation in hyperacute HIV infection or active infections as well as immunomodulatory treatments were excluded from the study to remove any potential confounding bias.
Ethics oversight	This is a study approved by the University of California San Francisco Committee on Human Research, SCOPE and OPTIONS cohorts at Zuckerberg San Francisco General Hospital (https://clinicaltrials.gov/ct2/show/NCT00187512). All participants were over 18 years old and provided written informed consent.

Note that full information on the approval of the study protocol must also be provided in the manuscript.

Field-specific reporting

Please select the one below that is the best fit for your research. If you are not sure, read the appropriate sections before making your selection.

☒ Life sciences ☐ Behavioural & social sciences ☐ Ecological, evolutionary & environmental sciences

For a reference copy of the document with all sections, see nature.com/documents/nr-reporting-summary-flat.pdf

Life sciences study design

All studies must disclose on these points even when the disclosure is negative.

Sample size	This study was designed to be observational so no sample size calculations were performed. A sample size of 10 participants per group (total N = 40) was elected according to the enrollment projections and demographics within the SCOPE and OPTIONS cohorts. It was determined that this number was sufficient to support the observational objectives while balancing the enrollment and budget constraints for this study.
Data exclusions	TREC quantification in distinct resting CD4 T cell subsets showed that the TTD/TEMRA fraction had up to 20% contamination with TN cells so the decision was made to exclude the TTD/TEMRA TREC data from the analysis.
Replication	The scarcity and uniqueness of clinical samples by nature prevented some experiments from being repeated. Quantifications of integrated HIV DNA were run in duplicates and successfully assayed.
Randomization	Participants were categorized into four groups according to the following criteria: ART initiated less than 100 days after acute infection and maintained for 1-3 years; ART initiated less than 100 days after acute infection and maintained for at least 7 years; ART initiated at least 3 years after acute infection and maintained for 1-3 years; and ART initiated at least 3 years after acute infection and maintained for at least 7 years. Additional covariates were tested in modeling analyses and no strong signals were found for experimental results based on age, sex, gender, or ART category.
Blinding	Clinical workers were solely knowledgeable about each participant's assignment into a given study group until all sample/data collection and analysis were completed.

Reporting for specific materials, systems and methods

We require information from authors about some types of materials, experimental systems and methods used in many studies. Here, indicate whether each material, system or method listed is relevant to your study. If you are not sure if a list item applies to your research, read the appropriate section before selecting a response.

Materials & experimental systems

n/a	Involved in the study
☐	☒ Antibodies
☒	☐ Eukaryotic cell lines
☒	☐ Palaeontology and archaeology
☒	☐ Animals and other organisms
☐	☒ Clinical data
☒	☐ Dual use research of concern

Methods

n/a	Involved in the study
☒	☐ ChIP-seq
☐	☒ Flow cytometry
☒	☐ MRI-based neuroimaging

Antibodies

Antibodies used	Live/Dead Fixable Aqua (Life Technologies, L34957 1:200 dilution); anti-CD3-FITC (Becton Dickinson, 561807, clone UCHT1, 1:200 dilution); anti-CD4-AlexaFluor700 (Becton Dickinson, 557922, clone RPA-T4 1:200 dilution); anti-CCR7-PE-Cyanine7 (Becton Dickinson, 557648, clone 3D12 1:100 dilution); anti-CD27-APC (Becton Dickinson, 337169, clone L128 1:100 dilution); anti-HLA-DR-APC H7 (Becton Dickinson, 561358, clone G46.6 1:100 dilution); anti-CD57-Brilliant Violet 421 (Becton Dickinson, 563896, clone NK1 1:200 dilution); anti-CD95-PE (Becton Dickinson, 555674, clone DX2 1:100 dilution); anti-CD45RA-ECD (Beckman Coulter, IM2711U, clone 2H4 1:100 dilution). Lot numbers were not tracked.
Validation	Antibodies were used per the manufacturer's recommendations unless a suitable concentration was determined by antibody titration prior to study start. All primary antibodies were validated in Human samples for use by flow cytometry. Validation statements can be found on manufacturer websites.

Clinical data

Policy information about clinical studies

All manuscripts should comply with the ICMJE guidelines for publication of clinical research and a completed CONSORT checklist must be included with all submissions.

Clinical trial registration	RAI116368A
Study protocol	This study was funded by NIAID under section [PAR14-247] - BASIC RESEARCH ON HIV PERSISTENCE (R01).
Data collection	Study participant recruitment and sample collection took place between 15 April 2015 and 19 February 2019. Data collection and analysis were finalized in January 2023.
Outcomes	The goals of this observational exploratory clinical study were described in the R01 award submitted to NIAID.

Flow Cytometry

Plots

Confirm that:

☒ The axis labels state the marker and fluorochrome used (e.g. CD4-FITC).

☒ The axis scales are clearly visible. Include numbers along axes only for bottom left plot of group (a 'group' is an analysis of identical markers).

☒ All plots are contour plots with outliers or pseudocolor plots.

☒ A numerical value for number of cells or percentage (with statistics) is provided.

Methodology

Sample preparation	PBMC were collected and isolated from peripheral blood draws and leukaphereses. Resting CD4 T cells were then enriched and sorted into subsets using flow cytometry.
Instrument	FACS ARIA II flow cytometer (BD Biosciences).
Software	Data collection was done using FACSDiva v8.0.1 (BD Biosciences), and data analysis was performed using FlowJo v8.7 (Tree Star).
Cell population abundance	Cells were sorted on a FACS ARIA II flow cytometer to >97% purity. Purity was assessed by running an aliquot of a post-sort cell subpopulation.
Gating strategy	From singlets PBMC (FSC-A/FSC-H and FSC/SSC gates), live (Aqua-) CD3+CD4+ lymphocytes were selected. We then sorted either the activated (HLA-DR+) fraction, or the following subpopulations within the resting (HLA-DR-) fraction according to their expression of phenotypic markers: naïve (TN, CD45RA+CCR7+CD27+CD57-CD95-), stem-cell memory (TSCM, CD45RA

+CCR7+CD27+CD57-CD95+), central memory (TCM, CD45RA−CCR7+CD27+), transitional memory (TTM, CD45RA−CCR7−CD27+), effector memory cells (TEM, CD45RA−CCR7−CD27−) and terminally-differentiated (TTD/TEMRA, CD45RA+CCR7-) cells.

☒ Tick this box to confirm that a figure exemplifying the gating strategy is provided in the Supplementary Information.
